# IL1RL1 is dynamically expressed on *Cbfb-MYH11*^+^ leukemia stem cells and promotes cell survival

**DOI:** 10.1038/s41598-018-38408-3

**Published:** 2019-02-11

**Authors:** Yiqian Wang, Lisa Richter, Michelle Becker, Catalina Amador, R. Katherine Hyde

**Affiliations:** 1 Department of Biochemistry and Molecular Biology, and Fred & Pamela Buffett Cancer Center, University of Nebraska Medical Center, Omaha, NE United States; 2 Department of Pathology and Microbiology, University of Nebraska Medical Center, Omaha, NE United States

## Abstract

Acute myeloid leukemia (AML) is often characterized by the presence of specific, recurrent chromosomal abnormalities. One of the most common aberrations, inversion of chromosome 16 [inv(16)], generates the fusion oncogene *CBFB-MYH11*. Previously, we used a mouse knock-in model to show that *Cbfb-MYH11* induces changes in gene expression and results in the accumulation of abnormal myeloid cells, a subset of which are enriched for leukemia stem cell (LSC) activity. One gene upregulated by *Cbfb-MYH11* encodes the cytokine receptor IL1RL1 (ST2). IL1RL1 and its ligand IL-33 are known regulators of mature myeloid cells, but their roles in AML are not known. Here, we use *Cbfb-MYH11* knock-in mice to show that IL1RL1 is expressed by cell populations with high LSC activity, and that the cell surface expression of IL1RL1 is dynamic, implying that the expression of IL1RL1 is not restricted to a specific stage of differentiation. We also show that treatment with IL-33 increased serial replating ability and expression of pro-survival proteins *in vitro*. Finally, we show that IL1RL1^+^ cells can survive chemotherapy better than IL1RL1^−^ cells *in vivo*. Collectively, our results indicate that IL1RL1 is dynamically expressed in *Cbfb-MYH11*^+^ leukemia cells and promotes their survival.

## Introduction

Acute myeloid leukemia (AML) is a hematopoietic malignancy often characterized by the presence of specific, recurrent chromosomal abnormalities^1^. One of the most common aberrations is inversion of chromosome 16 [inv(16)], which generates a fusion between the transcription factor gene, *CBFB*, and the gene for smooth muscle myosin heavy chain, *MYH11*^2,3^. Expression of *CBFB-MYH11* which encodes the fusion protein CBFβ-SMMHC, is the initiating event in inv(16) AML, but additional cooperating mutations are required for transformation to a frank leukemia. Common cooperating mutations include activating mutations in receptor kinases, such as KIT and fms like tyrosine kinase 3 (FLT3), or non-receptor kinases like RAS^4–8^. Although considered a prognostically favorable subtype of AML, approximately 50% of patients with inv(16) AML relapse and eventually die of their disease^9–12^. This is likely due to the persistence of leukemia stem cells (LSCs). LSCs are thought to be a small minority of cells that reside at the apex of a hierarchical differentiation scheme in leukemia and can both self-renew and generate non-self-renewing progenitor-like cells. LSCs are also thought to be mostly quiescent, allowing them to evade conventional chemotherapies which target primarily proliferating cells^13–16^.

Previously, a knock-in mouse model of inv(16) AML was established in which a conditional allele of *Cbfb-MYH11* is expressed from the endogenous *Cbfb* locus (*Cbfb*^+/*56M*^)^17,18^. We showed that expression of *Cbfb-MYH11* leads to changes in gene expression and an abnormal process of differentiation that culminates in a population of abnormal, immature myeloid cells expressing the cytokine receptor CSF2RB^17,19^. Using *in vivo* transplantations, we found that the presumably more immature, CSF2RB^−^ cells are enriched for LSC activity. We also identified a second cytokine receptor, IL1RL1 (ST2), which is highly expressed in *Cbfb-MYH11* expressing cells in both the CSF2RB^−^ and CSF2RB^+^ populations^19^. This raises the possibility that IL1RL1 could be expressed on LSCs and/or play a functional role in regulating their activity.

IL1RL1 is an IL-1 type receptor that is expressed on a subset of T cells and different types of mature myeloid cells, including mast cells, eosinophils, basophils, neutrophils and macrophages^20–22^. IL1RL1’s only known ligand is the cytokine IL-33. Binding of IL-33 to IL1RL1 on normal myeloid cells triggers a pro-inflammatory response, which can involve the release of additional cytokines, increased proliferation, and/or a block in apoptosis. Recent studies suggest that the IL1RL1/IL-33 pathway may be involved in malignant hematopoiesis as well. IL1RL1 is upregulated in chronic myeloid leukemia (CML) cells by the fusion protein BCR-ABL and treatment with IL-33 promotes resistance to the BCR-ABL inhibitor imatinib^23^. In addition, IL1RL1/IL-33 signaling exacerbates dysregulated myelopoiesis in mouse models of myeloproliferative neoplasms (MPN)^24^; however, its role in AML has not yet been demonstrated.

In the present study, we show that expression of the leukemogenic fusion gene *Cbfb-MYH11* induces expression of IL1RL1 prior to CSF2RB, implying that IL1RL1 marks an earlier stage of leukemia development. Thus, we tested whether IL1RL1, in conjunction with the hematopoietic stem/progenitor marker KIT, can be used to further enrich for LSCs in the CSF2RB^−^ population. Using limiting dilution transplantation assays (LDA), we found that CSF2RB^− ^IL1RL1^−^ KIT^+^, CSF2RB^−^ IL1RL1^+^ KIT^+^, and CSF2RB^−^ IL1RL1^+^ KIT^−^ cells showed considerable LSC activity *in vivo*. We also found that the rate of leukemia development correlated with the proliferation rate of each sub-population. Interestingly, we found that, regardless of the sub-population tested, the resultant leukemia had an immunophenotype similar to the original leukemia sample. We also show that activation of IL1RL1 signaling by IL-33 enhanced serial replating ability, while decreasing apoptosis and increasing viability. Finally, our preliminary data indicates that KIT expression cooperates with IL1RL1 in reducing apoptosis and inducing proliferation. Based on these findings, we propose that IL1RL1 cell surface expression is dynamic and contributes to leukemia cell survival.

## Results

### Expression of *Cbfb-MYH11* induces abnormal expression of IL1RL1

**Figure 1:**
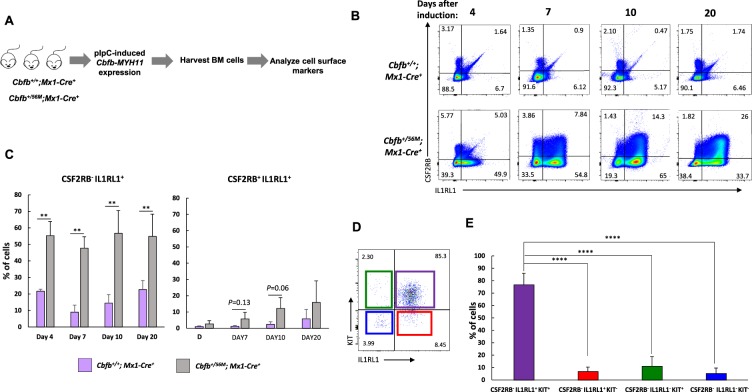
Dysregulated expression of cell surface marker expression by *Cbfb-MYH11*. (**A**) Schematic representation of experimental design. *Cbfb*^+/*56M*^*; Mx1-Cre*^+^ or *Cbfb*^+/+^*; Mx1-Cre*^+^ mice were injected with polyinosinic-polycytidylic acid (pIpC) to induce the expression of *Cbfb-MYH11*. Bone marrow cells were harvested, lineage negative (lin^−^) cells isolated, and stained with antibodies for IL1RL1 and CSF2RB. (**B**) Representative FACS plots showing the expression of CSF2RB and IL1RL1 at the indicated time points after the induction of *Cbfb-MYH11*. (**C**) Bar graph showing the percentages (%) of CSF2RB^−^ IL1RL1^+^ (left) and CSF2RB^+^ IL1RL1^+^ (right) cells at the indicated time points. (**D**) Representative IL1RL1 and KIT staining of CSF2RB^−^ cells from leukemic *Cbfb-MYH11*^+/*56M*^, *Mx1-Cre*^+^ mice. (**E**) Bar graph of IL1RL1 and KIT expression in CSF2RB^−^ population. N ≥ 3; **P < 0.01; ****P < 0.0001.

We showed previously that the expression of *Cbfb-MYH11* causes an abnormal differentiation process that culminates in cells expressing CSF2RB, and that the less differentiated CSF2RB^−^ population is enriched for LSCs^19^. Another cell surface marker upregulated by *Cbfb-MYH11* is IL1RL1. To examine if IL1RL1 could be a marker for less differentiated leukemia cells, we characterized the expression of IL1RL1 after induction of *Cbfb-MYH11* but before leukemia development. We used mice expressing a conditional allele of full-length *Cbfb-MYH11 (Cbfb*^+/*56M*^*)* paired with the inducible *Mx1-Cre* transgene^17^. *Cbfb*^+/+^*, Mx1-Cre*^+^ mice were used as control. Mice were treated with polyinosinic-polycytidylic acid (pIpC) to induce the expression of *Cbfb-MYH11*, and sacrificed on days 4, 7, 10, and 20 post-treatment. The lineage negative (lin^−^) bone marrow cells were stained for CSF2RB, IL1RL1, and the hematopoietic stem/progenitor cell marker KIT expression (Fig. 1A). We found that *Cbfb-MYH11* led to a significant increase of CSF2RB^−^ IL1RL1^+^ cells starting from day 4, as compared to control mice. Starting on day 7, we observed a smaller population of IL1RL1, CSF2RB double positive (CSF2RB^+^ IL1RL1^+^) cells, and this population continued to increase through day 20, but did not reach statistical significance as compared to the control mice (Fig. 1B,C). We did not observe changes in the expression of KIT in non-leukemic *Cbfb-MYH11*^+/*56M*^, *Mx1-Cre*^+^ mice as compared to control mice (Supplemental Fig. S1). To test if *Cbfb-MYH11* expression correlates with the abnormal cell surface marker expression, we examined the expression of *Cbfb-MYH11* in the lin^−^ bone marrow cells collected at 4, 7, and 10 days after pIpC treatment. We found that C*bfb-MYH11* was expressed at day 4 and its expression increased even further at day 7 (Supplemental Fig. S2), consistent with previous work^18^. This indicates that the changes in IL1RL1 and CSF2RB coincide with detectable expression of *Cbfb-MYH11*. Collectively, these results indicate that *Cbfb-MYH11* induces a population of CSF2RB^−^ IL1RL1^+^ first, which is followed by a population of cells expressing both markers. Our findings imply that IL1RL1 is associated with a population of cells at an earlier stage in the abnormal differentiation process induced by *Cbfb-MYH11*.

To determine whether IL1RL1 continues to be expressed in the LSC-enriched CSF2RB^−^ population after leukemic transformation, we stained leukemia cells isolated from *Cbfb*^+/*56M*^*, Mx1-Cre*^+^ mice for IL1RL1 and KIT. We found that the majority of leukemic CSF2RB^−^ cells express both IL1RL1 and KIT (Fig. 1D,E).

### CSF2RB^-^ sub-populations have different LSC activities ***in vitro*** and ***in vivo***

**Figure 2:**
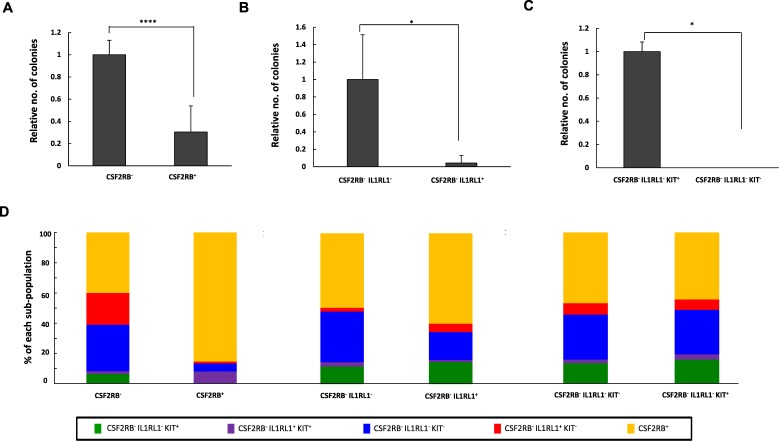
Colony forming cells are enriched in the CSF2RB^−^, IL1RL1^−^, KIT^+^ population. (**A–C**) Bar graphs showing the relative number of colonies observed from leukemic cells from 3 independent *Cbfb*^+/*56M*^*; Mx1-Cre*^+^ mice sorted for the indicated cell surface markers. (**D**) Bar graph showing the percentage of each sub-population in colonies observed from leukemic cells from 3 independent *Cbfb*^+/*56M*^*; Mx1-Cre*^+^ mice sorted for the indicated cell surface markers. N ≥ 3; *P < 0.05; ****P < 0.0001.

The observations that *Cbfb-MYH11* induces IL1RL1 earlier than CSF2RB, and that IL1RL1 is expressed on the majority of leukemic CS2FRB^−^ cells, raise the possibility that IL1RL1 can be used to further isolate LSCs. Before testing this, we confirmed that the leukemic CSF2RB^−^ population is enriched for colony-forming cells *in vitro*. We sorted leukemic cells isolated from 3 independent *Cbfb*^+/*56M*^*, Mx1-Cre*^+^ mice and performed colony-forming assays using equal numbers of CSF2RB^+^ and CSF2RB^−^ cells. We found that the CSF2RB^−^ cells produced significantly more colonies than did the CSF2RB^+^ cells, consistent with our previous *in vivo* results^19^ (Fig. 2A). Next, we tested whether IL1RL1 expression can further isolate LSCs. We found that the CSF2RB^−^ IL1RL1^−^ cells formed significantly more colonies than did CSF2RB^−^ IL1RL1^+^ cells, implying that LSCs are enriched within the CSF2RB, IL1RL1 double negative (CSF2RB^−^ IL1RL1^−^) population (Fig. 2B). Finally, we tested whether the hematopoietic stem and progenitor marker KIT can further enrich for LSCs. We found that significantly more colonies formed from CSF2RB^−^ IL1RL1^−^ KIT^+^ cells compared to CSF2RB^−^ IL1RL1^−^ KIT^−^ cells (Fig. 2C). To test whether the different populations gave rise to colonies with different phenotypes, we examined both the morphology and cell surface marker expression of the colonies that arose from each population. We found that colonies derived from the CSF2RB^−^ and CSF2RB^+^ populations had similar morphological appearance and gave rise to both CSF2RB^−^ and CSF2RB^+^ cells (Fig. 2D and Supplemental Fig. S3). Interestingly, the colonies from CSF2RB^−^ IL1RL1^−^, CSF2RB^−^ IL1RL1^+^, CSF2RB^−^ IL1RL1^−^ KIT^−^, and CSF2RB^−^ IL1RL1^−^ KIT^+^ sorted cells all gave rise to cells with similar immunophenotypic diversity (Fig. 2D). This finding implies that, despite the differences in colony-forming ability, the populations defined by CSF2RB, IL1RL1, and KIT may not be arranged in a strict hierarchical differentiation scheme. To determine if non-leukemic, healthy spleen cells could be giving rise to some of the observed colonies, we sorted spleen cells from wild type mice into CSF2RB^−^ IL1RL1^−^ KIT^+^ and CSF2RB^−^ IL1RL1^−^ KIT^−^ populations and plated the same number of cells as above in methylcellulose. We found that only the wild type CSF2RB^−^ IL1RL1^−^ KIT^+^ cells grew on methylcellulose, but they did not give rise to the distinct colonies seen with leukemic cells (data not shown). Collectively, these results indicate that the CSF2RB^−^ IL1RL1^−^ KIT^+^ population has increased colony-forming ability *in vitro*, but that each population gives rise to colonies with similar immunophenotypic and morphological characteristics.

**Figure 3:**
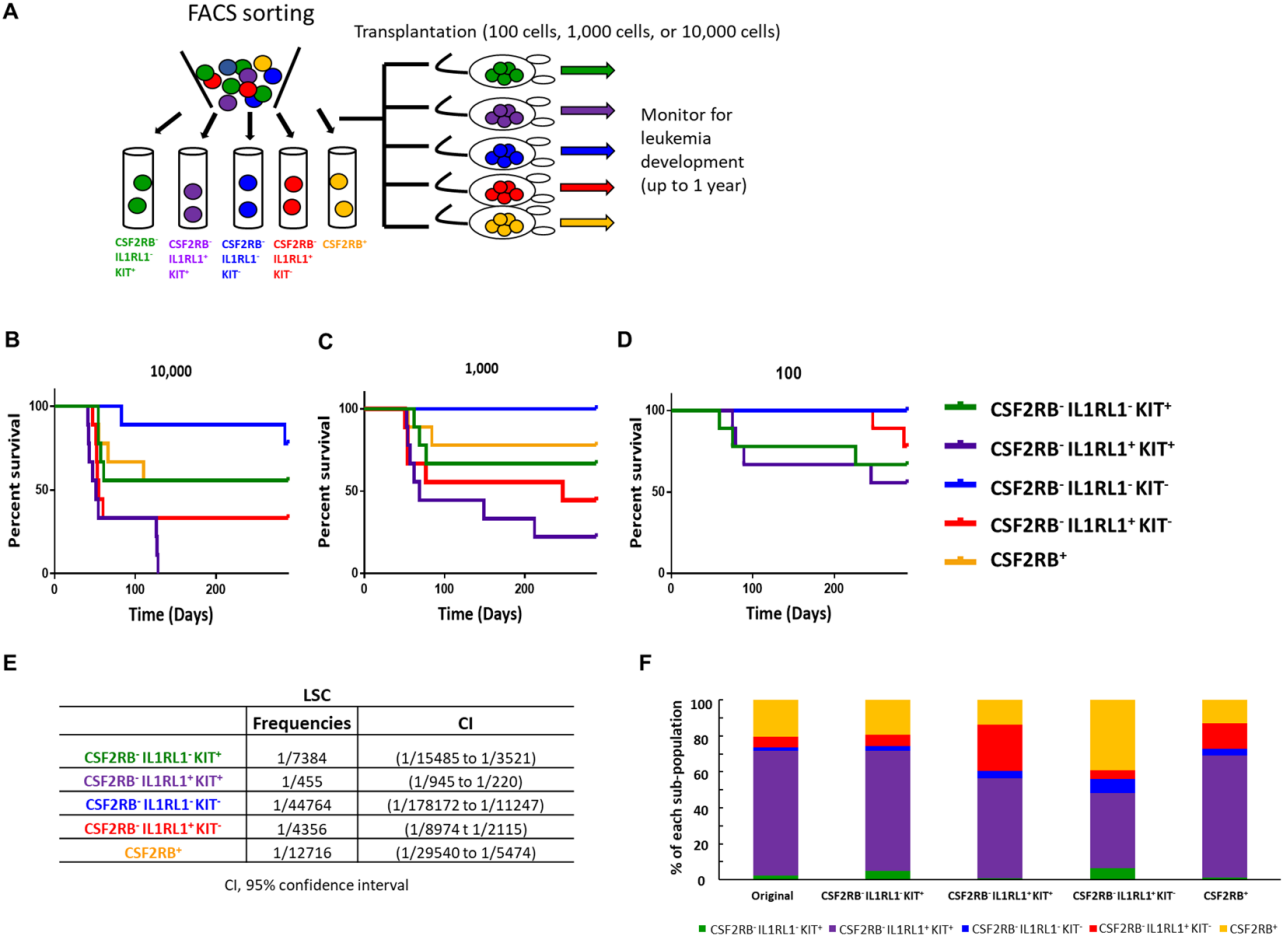
Multiple sub-populations have LSC activity *in vivo*. (**A**) Schematic representation of experimental design. Leukemia cells from 3 independent *Cbfb-MYH11*^+/*56M*^, *Mx1-Cre*^+^ mice were stained for CSF2RB, IL1RL1, and KIT and sorted into the indicated sub-populations. (**B–D**) Kaplan-Meier survival curves of mice transplanted with indicated number of cells from the indicated sub-populations. (**E**) Estimated frequency of LSCs and confidence interval for the indicated sub-populations. (**F**) Bar graph showing the percentage of each sub-population in the original leukemia sample, and in recipient mice transplanted with the indicated sub-population. For the log-rank test *P*-values see Supplementary Table S1.

To test whether IL1RL1 and KIT can be used to isolate cells with LSC activity *in vivo*, we performed LDA using leukemia samples from three independent *Cbfb-MYH11* expressing mice. Leukemia cells were then stained for CSF2RB, IL1RL1, and KIT, and sorted into five sub-populations: CSF2RB^−^ IL1RL1^−^ KIT^+^, CSF2RB^−^ IL1RL1^+^ KIT^+^, CSF2RB^−^ IL1RL1^−^ KIT^−^, CSF2RB^−^ IL1RL1^+^ KIT^−^, and CSF2RB^+^. To examine the sorting purity, we analyzed the surface marker expression of each sorted sub-population immediately after isolation via FACS. The purity in each sorted sub-population was more than 90% (Supplemental Fig. S4). 100, 1000, or 10,000 cells from each sorted sub-population were transplanted, and recipient mice were monitored for leukemia development (Fig. 3A). With the exception of CSF2RB^−^ IL1RL1^−^ KIT^−^ cells, we found that each sub-population caused leukemia at both the 1,000 and 10,000 cell doses, indicating that each of these sub-populations contains LSCs. Mice transplanted with the CSF2RB^−^ IL1RL1^+^ KIT^+^ cells showed the shortest average life span and highest penetrance of leukemia development with statistically significant differences in survival as compared to mice transplanted with CSF2RB^−^, IL1RL1^−^, KIT^+/−^, or CSF2RB^+^ cells at the 10,000 cell dose. Mice transplanted with CSF2RB^−^ IL1RL1^−^ KIT^−^ cells had the longest time to leukemia development and the lowest penetrance with statistically significant differences in survival as compared to mice transplanted with CSF2RB^−^, IL1RL1^−^, KIT^+/−^ cells at the 10,000 cell dose (Fig. 3B–D and Supplemental Table S1). Interestingly, the CSF2RB^−^, IL1RL1^−^, KIT^+^ population that showed enriched colony-forming ability *in vitro*, showed intermediate LSC activity *in vivo*. Based on these results, the CSF2RB^−^ IL1RL1^+^ KIT^+^ sub-population was calculated to contain the highest frequency of LSCs, which was between 10- and 100-fold higher than the other sub-populations, followed by CSF2RB^−^ IL1RL1^+^ KIT^−^ cells, and the CSF2RB^−^ IL1RL1^−^ KIT^−^ the lowest (Fig. 3E). The data suggests that CSF2RB^−^ IL1RL1^+^ cells are enriched for LSCs, especially those that are also expressing KIT. This is in contrast to our *in vitro* colony assay results and thus suggests that colony-forming ability does not strictly correlate with LSC activity *in vivo*.

To determine if the different sorted sub-populations cause leukemia with similar immunophenotypes, we performed cell surface staining on the leukemia cells from recipient mice. We observed that the expression of CSF2RB, IL1RL1, and KIT in recipient leukemia samples was similar to that of the original, donor leukemia samples, regardless of the sub-population transplanted (Fig. 3F). In addition, histological examination of blood smears from transplanted mice showed no differences in blast morphology (Supplemental Fig. S5). This result demonstrates that the different LSC sub-populations recapitulate the phenotypic diversity of the original sample, which is one of the defining criteria of LSCs.

One possible explanation to the unexpected finding that the CSF2RB^−^, IL1RL1^−^, KIT^+^ population was not the most enriched for LSCs is that some of the sorted sub-populations are contaminated with non-leukemic, healthy spleen cells. Two of the samples used for this analysis were derived from *Cbfb*^+/*56M*^, *Mx1-Cre*^+^ mice that also carry a transgene (*Gt(ROSA)26Sor*^*tm4(ACTB-tdTomato, -EGFP*/*Luo*^/*J* or *GFP*^+^) that expresses green fluorescent protein (GFP) after *Cre* excision. Because non-leukemic cells that undergo *Cre* excision are unlikely to persist for the 5–6 months required for the primary leukemia to develop, GFP expression can be used to distinguish leukemic cells from normal, healthy cells in this model. Using these mice, we found that the sub-population with the lowest percentage of GFP^+^ cells was the CSF2RB^−^ IL1RL1^−^ KIT^−^ population (average 92%,+/−11.3), with all other populations above 98% GFP^+^ (Supplemental Fig. S6). We also stained the spleen cells from wild type mice for CSF2RB, IL1RL1 and KIT and found that the majority of cells are negative for the three markers (CSF2RB^−^ IL1RL1^−^ KIT^−^) (Supplemental Fig. S7), which is consistent with our finding that this population contains the most non-leukemic, healthy cells. These results indicate that the only sub-population likely to contain significant numbers of non-leukemic, healthy cells is the CSF2RB^−^ IL1RL1^−^ KIT^−^, and that differences in LSC frequencies in the other populations is unlikely to be affected by normal spleen cells. To examine whether the differences in behavior among the different LSC sub-populations could be due to differences in *Cbfb-MYH11* expression, we performed RT-PCR for the fusion gene on sorted leukemia cells and found that that *Cbfb-MYH11* expression is similar among each of the sorted sub-population (Supplemental Fig. S8).

### LSC sub-populations have different rates of proliferation and sensitivities to chemotherapy

**Figure 4:**
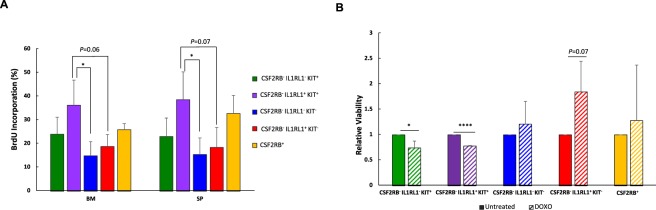
Different proliferation rates and chemotherapy sensitivities in LSC sub-populations. (**A**) Analysis of bromo-deoxyuridine (BrdU) incorporation in each LSC sub-population harvested from bone marrow (BM) and spleen (SP) of mice transplanted with GFP-expressing leukemic cells from 3 independent *Cbfb-MYH11*^+/*56M*^, *Mx1-Cre*^+^ mice. Mice were sacrificed 1 hour after a single BrdU injection. (**B**) Bar graph showing the relative viability of each LSC sub-population treated with 4 µM DOXO for 24 hours on OP-9 cells, as compared to the untreated cells. N ≥ 3; *P < 0.05; ****P < 0.0001.

In our transplantation study, we observed that the time taken to fatal leukemia development and leukemia penetrance was different among the LSC sub-populations, which may indicate a difference in proliferation rates, in addition to LSC frequency. To test this, we used leukemia cells from *Cbfb*^+/*56M*^, *Mx1-Cre*^+^ mice that also express green fluorescent protein (GFP), either from a lentivirus or from a transgene (*Cbfb*^+/*56M*^, *Mx1-Cre*^+^*, GFP*^+^). Mice were transplanted with GFP^+^ leukemia cells, monitored for leukemia development, and treated with bromodeoxyuridine (BrdU) prior to sacrifice. Cells from bone marrow and spleen were harvested and stained for cell surface markers, as well as BrdU incorporation. We found that the CSF2RB^−^ IL1RL1^+^ KIT^+^ population had the highest BrdU incorporation rate, which correlated with the fastest leukemia development after transplantation. The CSF2RB^−^ IL1RL1^−^ KIT^−^ population had the lowest BrdU incorporation rate, correlating with the longer latency required for leukemia development in mice transplanted with this sub-population (Fig. 4A).

Most frontline chemotherapies target proliferating cells. Therefore, one would predict that the most highly proliferative LSC sub-populations would be the most sensitive to chemotherapeutic drugs. To test this, we cultured each LSC sub-population on the bone marrow stromal cell line OP-9 and treated with doxorubicin (DOXO). We found that the two sub-populations with the highest relative proliferation rates, CSF2RB^−^ IL1RL1^−^ KIT^+^ and CSF2RB^−^ IL1RL1^+^ KIT^+^ showed a statistically significant decrease in viability as compared to the untreated cells. Interestingly, the less proliferative populations showed a trend towards increased relative viability when treated with DOXO, although this difference was not statistically significant (Fig. 4B). Together, these results imply that *Cbfb-MYH11* expressing LSCs can be highly proliferative and sensitive to chemotherapy *in vitro*.

### LSC markers are dynamic

**Figure 5:**
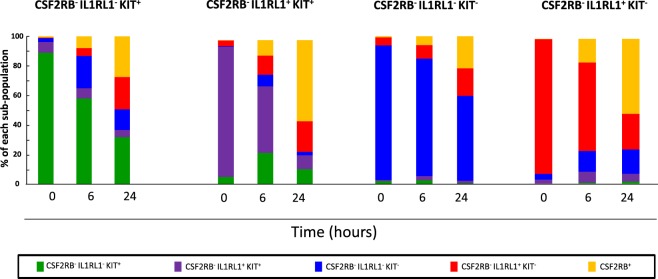
The expression of IL1RL1 and KIT is dynamic. Bar graph showing the expression of IL1RL1, KIT, and CSF2RB in leukemia cells from three independent *Cbfb-MYH11*^+/*56M*^, *Mx1-Cre*^+^ mice sorted for the indicated sub-populations, after co-culture with OP-9 cells for the indicated time. N = 3.

Our finding that all LSC sub-populations recapitulate the heterogeneity of the original leukemia sample implies that the sub-populations defined by CSF2RB, IL1RL1, and KIT are likely not to be organized in a strict hierarchical differentiation scheme. Rather, the expression of these markers may be dynamic. To test this possibility, we isolated each LSC sub-population based on their CSF2RB/IL1RL1/KIT expression and cultured them on OP-9 cells. We then stained cells for marker expression after 6 hours or 24 hours. We found that regardless of the initial sub-population sorted, the marker expression changed with time in culture. Interestingly, the CSF2RB^−^ IL1RL1^−^ KIT^−^ sub-population showed the most stable marker profile and the CSF2RB^−^ IL1RL1^−^ KIT^+^ sub-population was the most dynamic (Fig. 5). To exclude the possibility that the re-establishment of the marker profile was due to difference in survival, we compared the viability of each sorted sub-population at 0 hour and 24 hours. We found that there was no significant difference in viability among the LSC sub-populations after being cultured for 24 hours, indicating that each sub-population has similar rates of survival (Supplemental Fig. S9). This finding is consistent with other recent studies showing the plasticity of marker expression in LSCs and cancer stem cells from solid tumors^25,26^.

### IL1RL1 signaling promotes leukemia cell survival

**Figure 6:**
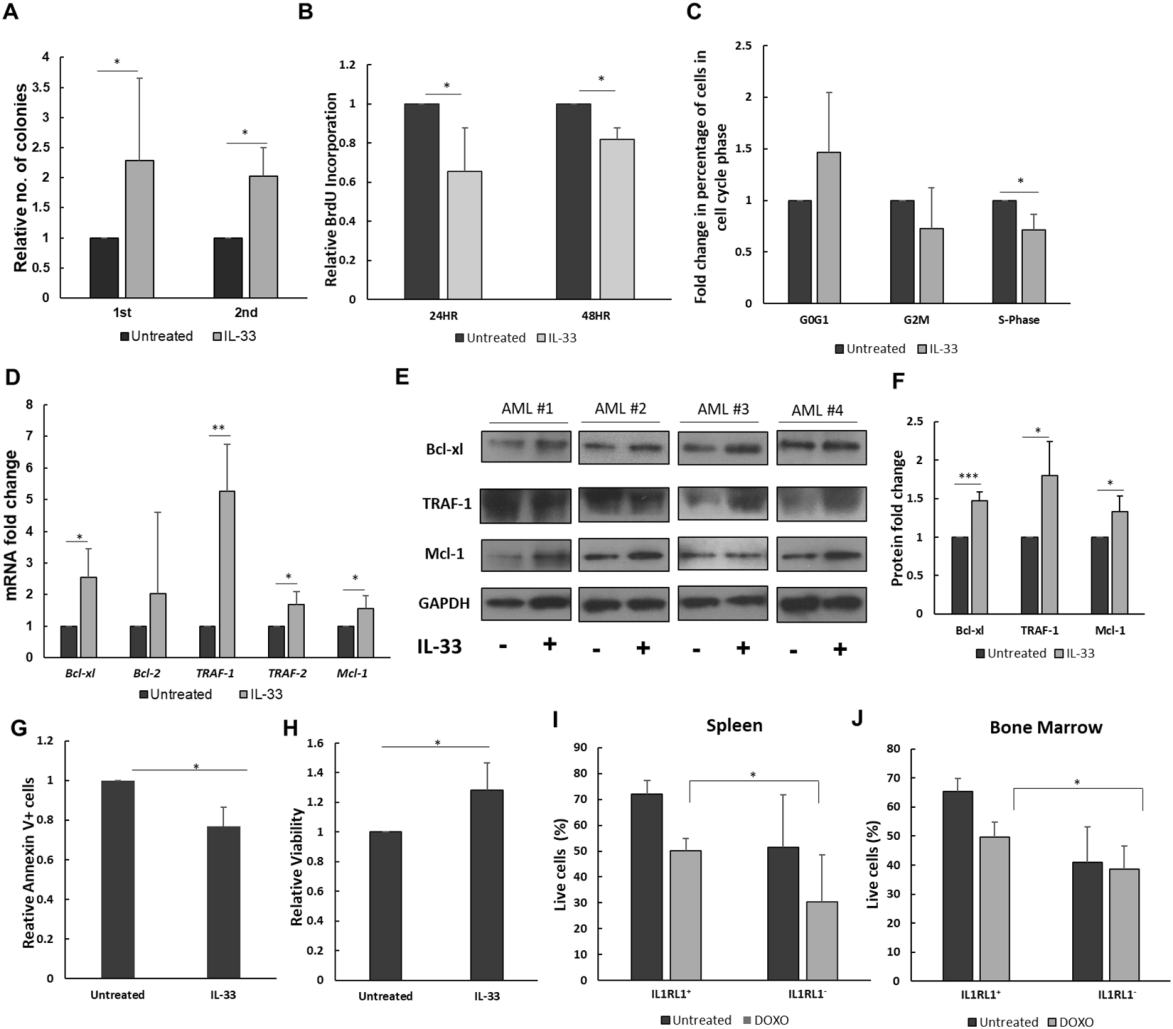
IL-33 promotes colony formation and survival of primary mouse *Cbfb-MYH11*^+^ leukemia cells. (**A**) Bar graph showing the relative number of colonies observed from equal numbers of *Cbfb-MYH11*^+/*56M*^, *Mx1-Cre*^+^ leukemia cells cultured in the presence or absence of IL-33 (100 ng/mL) in methylcellulose. Colonies were scored on day 14 (1st round), and then equal numbers of cells replated in methylcellulose, without additional IL-33 (2nd round). (**B**) Bar graph showing the relative BrdU incorporation in leukemia cells from *Cbfb-MYH11*^+/*56M*^, *Mx1-Cre*^+^ mice after culture for 24 and 48 hours in the presence or absence of IL-33 (100 ng/mL) compared to the untreated. Cells were pulse labeled with BrdU for 1 hour. (**C**) Bar graph showing the relative percentage of leukemia cells from *Cbfb-MYH11*^+/*56M*^, *Mx1-Cre*^+^ mice in the indicated phase of the cell cycle after culture for 24 hours in the presence or absence of IL-33 (100 ng/mL). (**D**) Bar graph showing the fold changes in mRNA expression of *Bcl-xl*, *Bcl-2*, *TRAF-1*, *TRAF-2*, and *Mcl-1* in leukemia cells from *Cbfb-MYH11*^+/*56M*^, *Mx1-Cre*^+^ mice cultured for 6 hours in the presence or absence of IL-33 (100 ng/mL). Relative expression levels were normalized to that of *Actb*. (**E**) Representative western blots of Bcl-xl, TRAF-1, Mcl-1, and the loading control GAPDH and (**F**) bar graph of relative protein levels in leukemia cells from four independent *Cbfb-MYH11*^+/*56M*^, *Mx1-Cre*^+^ mice cultured in the presence or absence of IL-33 (100 ng/mL). The membrane was cut into individual blots and incubated with indicated primary antibodies in separate dishes. After detection of Mcl-1, the blot was stripped and probed sequentially with antibody to GAPDH. (**G**) Bar graph showing the relative Annexin V staining and (**H**) viability in leukemia cells from *Cbfb-MYH11*^+/*56M*^, *Mx1-Cre*^+^ mice after culture for 24 hours in the presence of IL-33 (100 ng/mL) or combined with anti-IL1RL1 antibody (1 µg/mL) compared to the untreated cells. (**I**) Bar graphs showing the percentage of live cells from spleen and (**J**) bone marrow within IL1RL1^+^ and IL1RL1^−^ populations 48 hours after DOXO administration. For full scans of western blots from panel (**E**) see Supplemental Fig. S11. N ≥ 3; *P < 0.05; **P < 0.01.

**Figure 7:**
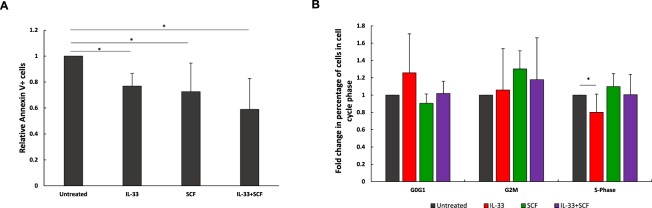
KIT cooperates with IL1RL1 in inhibiting apoptosis and inducing proliferation. (**A**) Bar graph showing the relative Annexin V staining in leukemia cells from *Cbfb-MYH11*^+/*56M*^, *Mx1-Cre*^+^ mice after culture for 24 hours in the presence of IL-33 (100 ng/mL), SCF (100 ng/mL) or in combination, compared to the untreated cells. (**B**) Bar graph showing the relative percentage of leukemia cells from *Cbfb-MYH11*^+/*56M*^, *Mx1-Cre*^+^ mice in the indicated phase of the cell cycle after culture for 24 hours in the presence of IL-33 (100 ng/mL), SCF (100 ng/mL) or in combination, compared to the untreated cells. N = 3. *P < 0.05.

The findings described above imply that the IL1RL1 and KIT cell surface expression likely does not identify populations arranged in a strict hierarchical differentiation scheme. However, their expression does define populations with distinct growth characteristics, implying that signaling through these receptors may regulate LSC activity. KIT is a well-established regulator of leukemia cell survival and activating mutations in KIT cooperate with *Cbfb-MYH11* during leukemia development^8^. However, whether IL1RL1 similarly regulates AML cells is not known. To test this hypothesis, we treated mouse leukemia cells with the IL1RL1 ligand, IL-33, and performed serial replating experiments. Equal numbers of leukemia cells were plated in methylcellulose in the presence or absence of IL-33. After 14 days of culture, colonies were counted, and equal numbers of cells were serially replated in methylcellulose without further addition of IL-33. At both the 1st and 2nd plating, we found that IL-33 led to significantly increased number of colonies (Fig. 6A). To confirm that the effect of IL-33 is mediated by IL1RL1, we treated cells with IL-33 and a blocking antibody against IL1RL1. We found that the enhanced colony-forming ability induced by IL-33 is blocked by the anti-IL1R1L antibody (Supplemental Fig. S10). These results indicate that IL-33/IL1RL1 signaling promotes colony-forming ability in *Cbfb-MYH11* expressing leukemia cells. To test if the increase in colony number is related to increased proliferation, we measured BrdU incorporation and cell cycle status on leukemia cells treated with IL-33. We found that IL-33 significantly decreased BrdU incorporation, as well as the percentage of cells in S-phase (Fig. 6B,C), indicating that IL-33 does not induce proliferation in *Cbfb-MYH11*^+^ leukemia cells. To test if increased cell survival could mediate IL-33’s effect on colony-forming ability, we performed quantitative reverse-transcription PCR (qRT-PCR) for genes known to be induced by IL-33 in normal myeloid cells: *Bcl-xl*, *Bcl-2*, *TRAF-1*, *TRAF-2*, and *Mcl-1*^27,28^. After 6 hours of stimulation with IL-33, we observed significantly increased expression of *Bcl-xl*, *TRAF-1*, *TRAF-2*, and *Mcl-1*, but not *Bcl-2* in leukemia cells (Fig. 6D). By western blot, we found that the protein levels of Bcl-xl, TRAF-1, and Mcl-1 were less dramatically, but statistically significantly increased in IL-33-treated cells, as compared to untreated cells (Fig. 6E,F and Supplemental Fig. S11). To test if IL-33 treatment protects cells from apoptosis, we stained leukemia cells with Annexin V. We found that IL-33-treated cells had statistically significant lower Annexin V staining, and increased viability, as compared to the untreated cells, and the anti-apoptotic effect of IL-33 was blocked by the addition of anti-IL1RL1 antibody (Fig. 6G,H). The decreased proliferation and apoptosis caused by IL-33 raises the possibility that signaling through IL1RL1 may be involved in chemoresistance. To test this possibility, we examined if there is a difference in the response between IL1RL1^+^ and IL1RL1^−^ leukemia cells to DOXO *in vivo*. Leukemic *Cbfb*^+/*56M*^, *Mx1-Cre*^+^*, GFP*^+^ mice were intraperitoneally injected with DOXO and leukemia cells from spleen and bone marrow were isolated 48 hours later. We found that the viability of IL1RL1^+^ cells is significantly higher than IL1RL1^−^ cells from both spleen and bone marrow, indicating that IL1RL1^+^ cells are less sensitive to killing by DOXO as compared to IL1RL1^−^ cells *in vivo* (Fig. 6I,J). This result is seemingly in contrast to our *in vitro* assay, where CSF2RB^−^ IL1RL1^+^ KIT^+^ is highly sensitive to DOXO treatment while CSF2RB^−^ IL1RL1^+^ KIT^−^ population is less sensitive in the presence of DOXO. To test whether KIT signaling influences the role of IL1RL1 in cell survival, we treated leukemic cells from 3 independent *Cbfb*^+/*56M*^*; Mx1-Cre*^+^ mice with IL-33, stem cell factor (SCF, the ligand for KIT) or the combination of IL-33 and SCF in culture for 24 hours and measured apoptosis and cell cycle status. We found that treatment with SCF or IL-33 caused a significant decrease in apoptosis, as compared to the untreated cells. The combination of IL-33 and SCF together showed a trend towards decreased apoptosis, although this difference was not statistically significant as compared to treatment with either cytokine alone (Fig. 7A). Treatment with SCF caused a trend towards an increase in cells in S phase, although this was not statistically significant. Interestingly, the combination of SCF and IL-33 did not show a decrease in S phase as observed with IL-33 alone (Fig. 7B), which may explain the different sensitivities of the CSF2RB^−^ IL1RL1^+^ KIT^−^ and CSF2RB^−^ IL1RL1^+^ KIT^+^ populations to DOXO. This is consistent with previous work showing that KIT and IL1RL1 cooperate to induce proliferation in mast cells^29^. These findings indicate that the IL1RL1/IL-33 signaling axis promotes leukemia cell colony-forming ability and resistance to DOXO, perhaps in part through the upregulation of pro-survival genes and a consequent block in apoptosis.

## Discussion

Previously, we showed that expression of the leukemogenic fusion gene *Cbfb-MYH11* induces changes in gene expression and causes aberrant myeloid differentiation^19^. Among the most differentially expressed genes are the cytokine receptors CSF2RB and IL1RL1. We also found that expression of CSF2RB is associated with decreased LSC activity, implying that CSF2RB signaling is likely not a major regulator of *Cbfb-MYH11*^+^ LSCs. However, the role of IL1RL1 was not known. In more recent work, IL1RL1 has been shown to be important in other myeloid malignancies, including CML and myeloproliferative neoplasms (MPN)^23,24^. Thus, in this report we examined the expression of IL1RL1 in the LSC-enriched CSF2RB^−^ population, and the effect of the IL1RL1 ligand, IL-33, on *Cbfb-MYH11* expressing leukemia cells.

By performing LDAs in congenic recipient mice, we found that multiple sub-populations in the CSF2RB^−^ fraction had relatively high rates of proliferation and considerable LSC activity. This is in contrast to most common models of LSCs which posit that LSCs are mostly quiescent and restricted to a single HSC-like population^13–16^. However, these models are primarily based on observations using human patient cells grown *in vitro* or in immunocompromised mouse models. It is possible that these experimental conditions only allow for the growth of a limited subset of LSCs, leading to an underestimation of the full spectrum of cells with LSC activity. In fact, we found that CSF2RB^−^ IL1RL1^+^ cells showed significantly impaired colony-forming ability when grown *in vitro*, despite their robust ability to cause leukemia when transplanted into recipient mice. The observed discrepancy between *in vitro* and *in vivo* experiments indicates that *in vitro* activities of leukemia cells may not reliably predict their *in vivo* phenotypes. This implies that cytokines and/or growth factors in the microenvironment are likely to play a critical role in regulating the leukemia stem cell activity. Studies using other mouse models have similarly shown that LSCs can be highly proliferative and are not restricted to an HSC-like population^30–32^. Collectively, these studies imply that LSCs are more diverse and less quiescent than what has been indicated by *in vitro* or xenograft assays. Furthermore, our finding that LSC activity correlates with proliferation in *Cbfb-MYH11*^+^ leukemia may in part explain the favorable response of inv(16) AML patient to traditional chemotherapy.

We also found that KIT and IL1RL1 did not identify a single LSC population that was clearly at the apex of a hierarchical differentiation scheme. Rather, we found that, aside from the CSF2RB^−^ IL1RL1^−^ KIT^−^ population, which had very little LSC activity, each of the sorted sub-populations were able to recapitulate the immunophenotypic diversity of the original leukemia sample in spite of their different growth characteristics and latencies *in vivo*. Currently, we can only speculate about the reasons why different populations have different growth kinetics. Presumably, it is related to signaling through the specific cytokine receptors expressed, which may in turn affect gene expression. We found that there is no significant difference in the expression of *Cbfb-MYH11* among each LSC sub-population, indicating that the differences in behavior among the different LSC sub-populations are not due to differences in expression of the fusion protein.

We found that the immunophenotype of the sorted sub-populations was altered in as short as 6 hours in culture. This indicates that the expression of KIT and IL1RL1 is highly dynamic and may not be restricted to a specific stage of differentiation. Currently, we can not distinguish whether this plasticity is due to inherent dynamic cell surface expression of these receptors, or whether de-differentiation of the different sub-populations allows them to give rise to cells with more stem-cell like characteristics. In fact, the presumed most HSC- like CSF2RB^−^ IL1RL1^−^ KIT^+^ population showed a trend towards the most plasticity, which may imply that expression of these markers may be related to differentiation, even if their expression is not strictly confined to a specific stage. However, our findings are similar to recent work in B-cell precursor acute lymphoblastic leukemia (BCP-ALL) demonstrating that expression of the human HSPCs markers CD34 and CD38 is highly plastic at the single cell level and recent work in AML showing a non-hierarchical relationship between CD34^+^ and CD34^−^ LSCs^25,33^.

Currently, we can only speculate as to the molecular mechanisms underlying the observed plasticity. It is possible that the expression of these cell surface marker changes in response to different environmental stimuli. In fact, the dynamic expression of KIT and IL1RL1 in leukemia cells suggests that these receptors may be regulating cell processes other than differentiation. KIT is known to be important specifically in inv(16) AML. Activating mutations in KIT are found frequently in inv(16) patient samples and are known to cooperate with *Cbfb-MYH11* during leukemia development^6,8,34–36^. Here we show that signaling through the IL1RL1 receptor also plays a role in leukemia cell survival. We found that treatment of *Cbfb-MYH11*^+^ leukemia cells with exogenous IL-33, the only known IL1RL1 ligand, reduced apoptosis and proliferation, and increased serial replating ability. This implies that IL1RL1 is important for LSC survival and self-renewal, which may partially explain the discrepancy between *in vitro* and *in vivo* assays for LSCs. These findings are similar to what has been shown in CML where IL-33 promotes LSC survival^23^. Importantly, the anti-apoptotic effect by IL-33 is subtle compared to the effect on colony-forming ability, implying that IL-33 is likely to affect other pathways in addition to apoptosis. Finally, we found that IL1RL1^+^ *Cbfb-MYH11*^+^ cells are less sensitive to treatment with doxorubicin, implying that IL-33/IL1RL1 signaling may promote cell survival in response to chemotherapy.

Taken together, our studies demonstrate that IL1RL1 is a potentially important regulator of *CBFB-MYH11*^+^ LSCs. Further, our results contribute to a growing body of evidence that LSCs may be more diverse than previously thought.

## Methods

### Ethics Statement

This study was performed in accordance with the guidelines established by the Guide for the Care and Use of Laboratory Animals at the National Institutes of Health. All experiments involving mice were approved by the Institutional Animal Care and Use Committee at the University of Nebraska Medical Center.

### Animals

Wild-type C57BL6/129SvEv mice (Taconic) were used as transplant recipients. Gt(ROSA)26Sor^tm4(ACTB-tdTomato, -EGFP/Luo^/J were obtained from Jackson Laboratory, and bred with *Cbfb-MYH11*^+/*56M*^, *Mx1-Cre*^+^ mice^17,37,38^. Mice were housed under specific pathogen-free conditions and sacrificed for the analysis at the indicated times. Mice were genotyped by polymerase chain reaction (PCR) using tail-snip DNA, as described previously^18,19,37^. Mice were treated with a single 200 µg dose of polyinosine-polycytidylic acid (pIpC) by intraperitoneal injection to induce *Cbfb-MYH11* expression, and monitored for leukemia development, as described previously^18,19^. Transplantation was performed by retro-orbital injection into sub-lethally irradiated congeneic recipients (600 cGy, 1–2 hours prior to transplantation). Recipient mice were monitored daily for any abnormal behavior or physiologic changes. Leukemia development was monitored up to one year. Doxorubicin (Pfizer Inc.) was reconstituted with phosphate-buffered saline (PBS) and filtered. Leukemic mice were administered an intraperitoneal injection of 2 mg/kg doxorubicin and sacrificed 48 hours later. All animals used and the procedures performed in this study were approved by the University of Nebraska Medical Center Institutional Animal Care and Use Committee.

### Tissue culture

Mouse leukemia cells isolated from the spleens of sick animals were cultured in RPMI 1640 (ATCC), supplemented with 20% fetal bovine serum (FBS) ES-Qualified (Life Technologies), 2mM L-Glutamine, and 1% penicillin-streptomycin. Media was supplemented with cytokines at the indicated concentration: 10 ng/mL IL-3 (Peprotech), 10 ng/mL IL-6 (Peprotech), 20 ng/mL SCF (Peprotech). Where indicated, IL-33 (Peprotech) was used at 100 ng/mL, anti-IL1RL1 antibody (R&D systems) used at 1 µg/mL, and SCF (Peprotech) used at 100 ng/mL. OP-9 cells were cultured in α-MEM (Gibco) with 20% FBS (Hyclone). GFP^+^ mouse leukemia cells isolated from the spleens of sick animals were cultured on a confluent layer of irradiated OP-9 cells in media supplemented with 57 µM β-mercaptoethanol (Sigma). HEK293T cells were maintained in DMEM medium (Corning) supplemented with 10% FBS, 2mM L-Glutamine and 1% penicillin-streptomycin. For lentivirus production and infection, HEK293T cells were transfected with third generation lentiviral plasmids which contain GFP and viral supernatant was collected 48 hours post-transfection. The viral supernatant was incubated with mouse leukemia cells isolated from the spleen of sick animals, supplemented with cytokines as described above with the addition of 8 μg/mL polybrene. Cells were spinfected at 2,000 rpm for 90 minutes, followed by a 6-hour incubation and a second spinfection. 48 hours after the start of transduction, cells were sorted using the GFP signal. Colony assays were performed using sorted leukemia cells suspended in Methocult 3434 (StemCell Technologies) according to the manufacturer’s protocol. For each experiment with a single leukemia sample, equal number of cells from each sub-population were plated in triplicate. Because the number of cells we were able to sort differed among leukemia samples, the number of cells plated varied across experiments. After 14 days in culture, plates were scored for colony number and replated as indicated. All cells were cultured in a 37 °C humidified atmosphere containing 5% CO_2_.

### FACS staining and sorting

Lineage-negative pre-leukemic cells were isolated from the bone marrow of pIpC-treated mice using the EasySep™ negative selection mouse hematopoietic progenitor enrichment cocktail (StemCell Technologies) according to the manufacturer’s instructions. Cells were stained with PE conjugated anti-CSF2RB (BD Pharmingen), PER-CP5.5 conjugated anti-IL1RL1 (BioLegend), and APC conjugated anti-KIT (BD Biosciences).

For transplantation studies of sorted LSC sub-populations, mouse leukemia cells isolated from the spleens of sick mice were stained with PE conjugated anti-CSF2RB (BD Pharmingen). CSF2RB^−^ cells were enriched and selected on an autoMACS Pro Separator (Miltenyi) using PE-microbeads and column per manufacturer’s instructions. Cells were further stained with APC conjugated anti-KIT (BD Biosciences) and PER-CP5.5 conjugated anti-IL1RL1 (BioLegend). Cellular viability was determined by staining with 4′,6-diamidino-2-phenylindole (DAPI). Cell proliferation was determined by BrdU incorporation using the BrdU Flow Kit (BD Pharmingen) according to the manufacturer’s instructions.Cells were pulse labeled with BrdU for 1 hour, and mice were sacrificed 1 hour after a single BrdU injection. Cell cycle was determined by propidium iodide (PI) staining after cells were fixed with 70% ethanol. Cells were analyzed using BD LSRII flow cytometer (BD Biosciences) and sorted using BD FACSAria (BD Biosciences) equipped with FACSDiva software (Becton Dickinson). FACS data was analyzed by FlowJo software (Tree Star) and ModFit software (Verity Software).

### Histological staining

Sorted cells were affixed to Surgipath Apex Superior Adhesive Slides (Leica MICROSYSTEMS) using a Shandon Cytospin III cytocentrifuge for 5 minutes at 800 rpm. Slides were stained using Protocol Hema 3 Wright-Giemsa stain (Thermo Fisher Scientific) according to the manufacturer’s protocol and were examined using an Olympus BX51 microscope at 100X magnification.

### Quantitative RT-PCR analysis

Total RNA was isolated from cells using Trizol reagent (Invitrogen), according to the manufacturer’s specifications, synthesized into cDNA using reverse transcription PCR (GE Health). Real-time quantitative analysis (qRT-PCR) was performed using Maxima SYBR Green/ROX qPCR Master Mix (Thermo Fisher Scientific) with specific primers for *Actb*, *Bcl-xl*, *Bcl-2*, *TRAF-1*, *TRAF-2*, and *Mcl-1* (Integrated DNA Technologies). The real-time PCR was run in a StepOnePlus Real-Time PCR System (Invitrogen). Expression levels of genes were normalized to *Actb* mRNA, and IL-33-stimulated versus unstimulated groups were compared applying the 2^−ΔΔCT^ method. Primers for qRT-PCR were described previously and shown in Supplemental Table S2^27,39,40^.

### Western blot analysis

Mouse leukemia cells isolated from the spleen of sick animals were lysed with RIPA buffer (50 mM Tris-HCl [pH 8.0], 150 mM NaCl, 1% Triton, 0.5% deoxycholate, and 0.1% SDS), supplemented with a protease inhibitor (Roche). Samples were run on 4–12% Bis-Tris gel (Invitrogen), transferred to PVDF membrane (NOVEX). The membrane was incubated with the primary antibody overnight, followed by 1-hour incubation with horseradish peroxidase (HRP)-conjugated anti-rabbit or anti-mouse secondary antibodies at room temperature, washed and incubated with chemiluminescence (ECL) reagent (Thermo Fisher Scientific) and then exposed to film. Densitometric analysis was performed using Image J software (National Institutes of Health). The relative protein level was normalized to the corresponding GAPDH protein level. The following primary antibodies were used for western blotting: Bcl-xl (sc-8392, Santa Cruz Biotechnology), TRAF-1 (sc-6253, Santa Cruz Biotechnology), Mcl-1 (sc-819, Santa Cruz Biotechnology), GAPDH (6C5, Ambion).

### Statistical analysis

The Kaplan-Meier curves of overall survival (OS) were plotted, and log-rank (Mantel-Cox) test was performed by Graph Pad Prism 7 for Windows (Graph Pad Software Inc.). Data are represented as mean values ± standard deviation. The significance of difference between two groups was determined by Student *t* tests in Excel (Microsoft). *P* values < 0.05 were considered statistically significant. The LSC frequency was calculated and plotted using ELDA software (bioinf.wehi.edu.au/software/elda/)^41–43^.

## Supplementary information

Supplemental Figures
