# Supplementary material for: IL1RL1 is dynamically expressed on Cbfb-MYH11+ leukemia stem cells and promotes cell survival
Source: Sci Rep. 2019 Feb 11;9:1729. doi: 10.1038/s41598-018-38408-3 (PMC6370767; doi:10.1038/s41598-018-38408-3)
Supplement: Supplementary file 1 — Supplemental Figures [file 41598_2018_38408_MOESM1_ESM.pdf]

IL1RL1 is dynamically expressed on *Cbfb-MYH11*$^+$ leukemia stem cells and promotes cell survival

Yiqian Wang[1], Lisa Richter[1], Michelle Becker[1], Catalina Amador[2], and R. Katherine Hyde[1*]

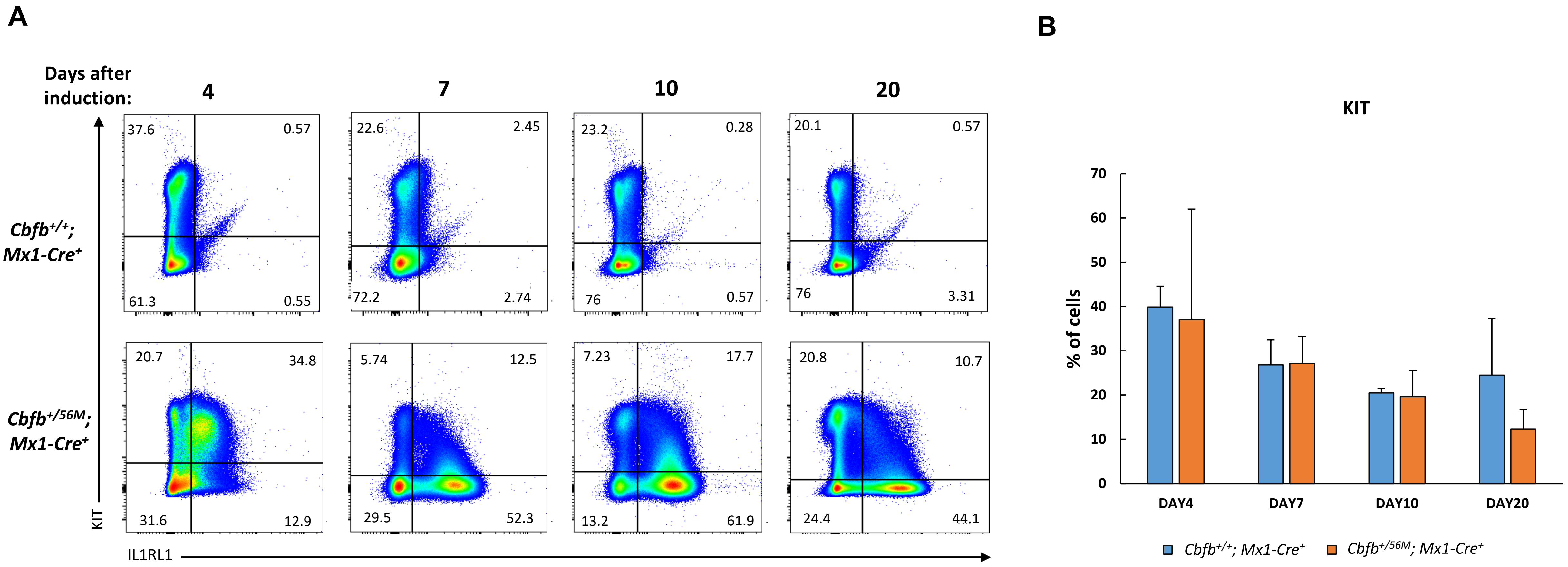

Supplemental Figure S1. The expression of KIT after pIpC treatment.

(**A**) Representative FACS plots showing the expression of KIT and IL1RL1 at the indicated time points after the induction of *Cbfb-MYH11*. (**B**) Bar graph showing the percentages (%) of KIT^{+} cells at the indicated time points. N≥3.

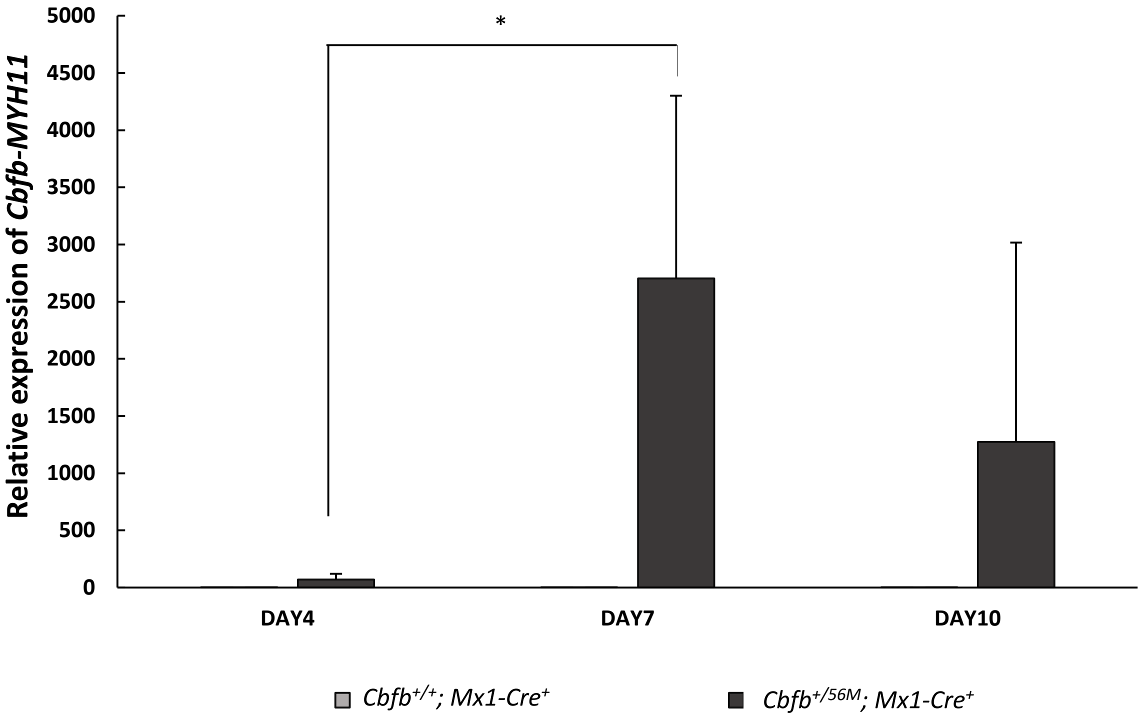

Supplemental Figure S2. Expression of *Cbfb-MYH11* after pIpC treatment.

Bar graph showing the qRT-PCR analysis of mRNA expression of *Cbfb-MYH11* allele 4, 7 and 10 days after induction of *Cbfb-MYH11* by pIpC. N=3; **P*<.05.

CSF2RB$^-$ **CSF2RB$^+$**

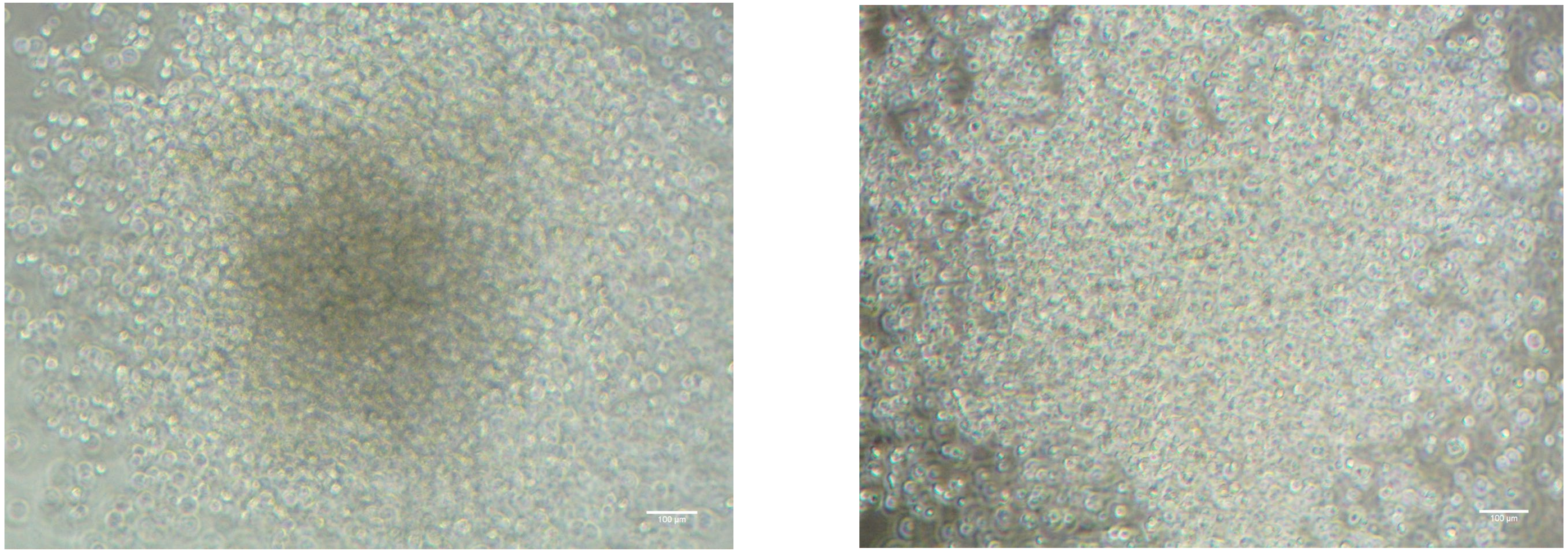

Supplemental Figure S3. Methylcellulose colonies derived from different sub-populations do not have difference in morphology.

Representative photomicrographs of methylcellulose colonies derived from CSF2RB$^-$ and CSF2RB$^+$ cells after 14 days of culture are shown. Scale bar:100 µm.

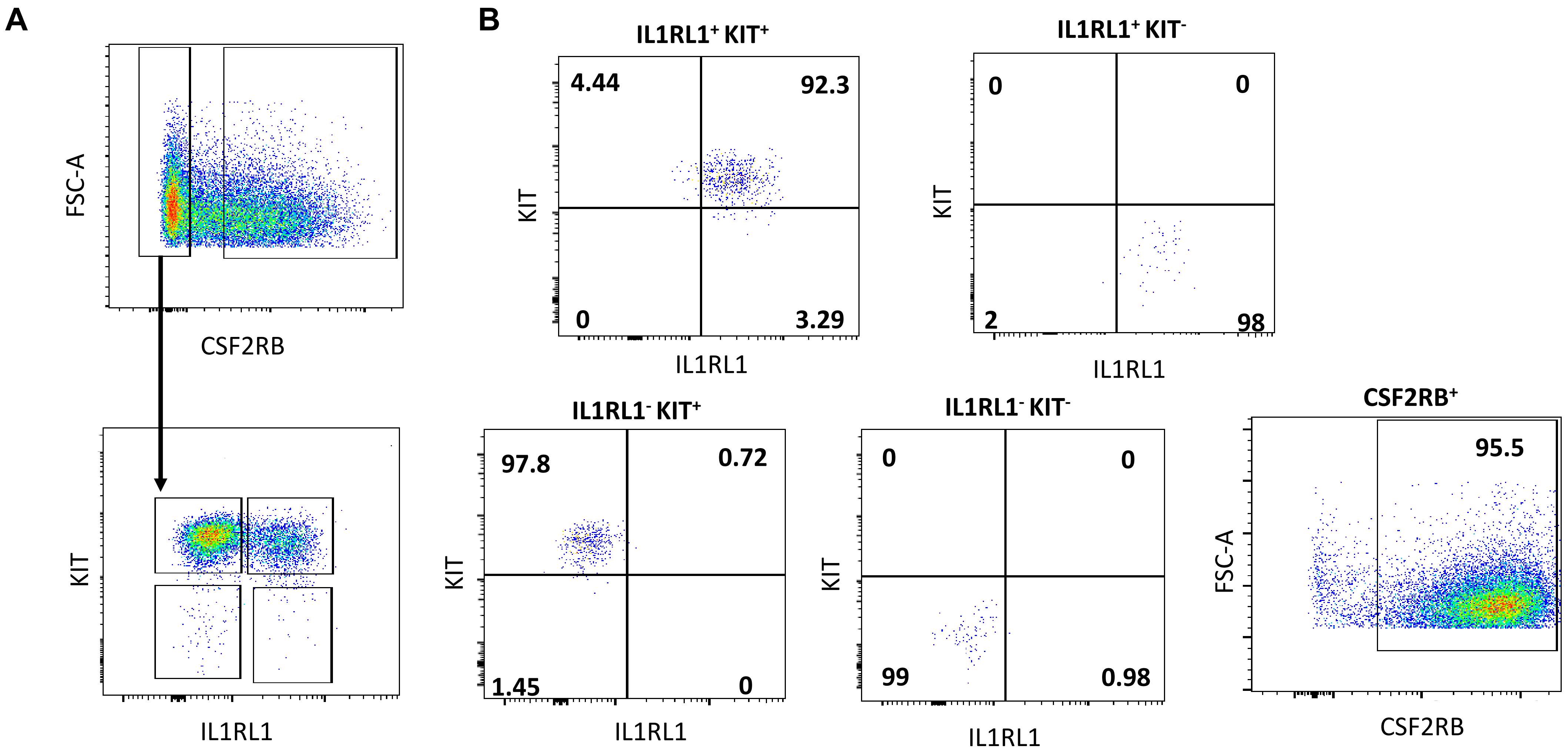

Supplemental Figure S4. Sorting strategy and post-sort analysis of each sub-population.

(**A**) Mouse leukemia cells were stained with anti-CSF2RB, anti-IL1RL1, and anti-KIT antibodies. Each sub-population was then sorted on a BD FACS Aria. Dot plots quantitate percentages of cells shown in each gate. (**B**) Representative FACS plots of post-sort analysis of the sorted sub-populations. The purity was greater than 90%. Data are from one of the three independent experiments.

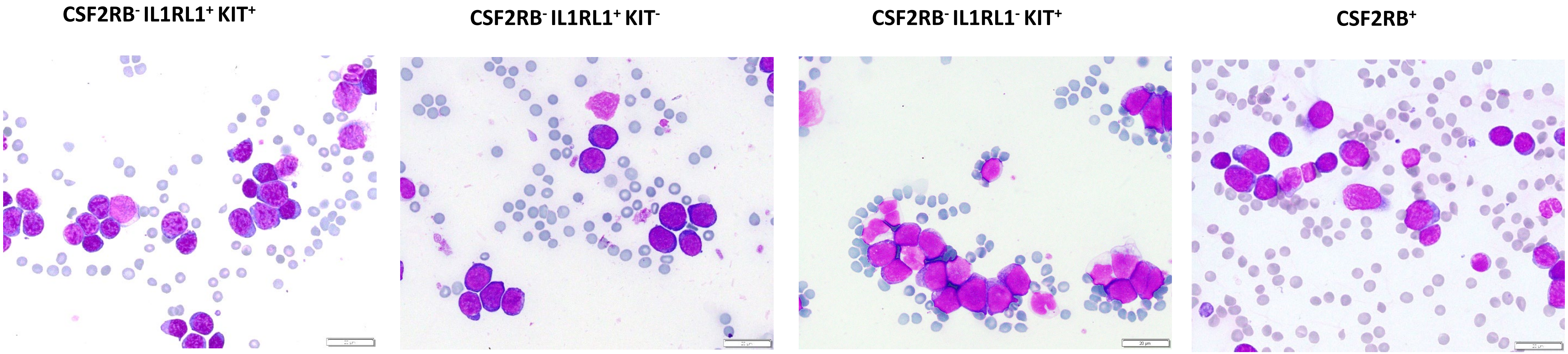

Supplemental Figure S5. Leukemia cells from each of the sorted sub-populations have similar appearances.

Representative Wright-Giemsa staining of peripheral blood smears from mice transplanted with the indicated LSC sub-populations. Scale bar: 20 μm.

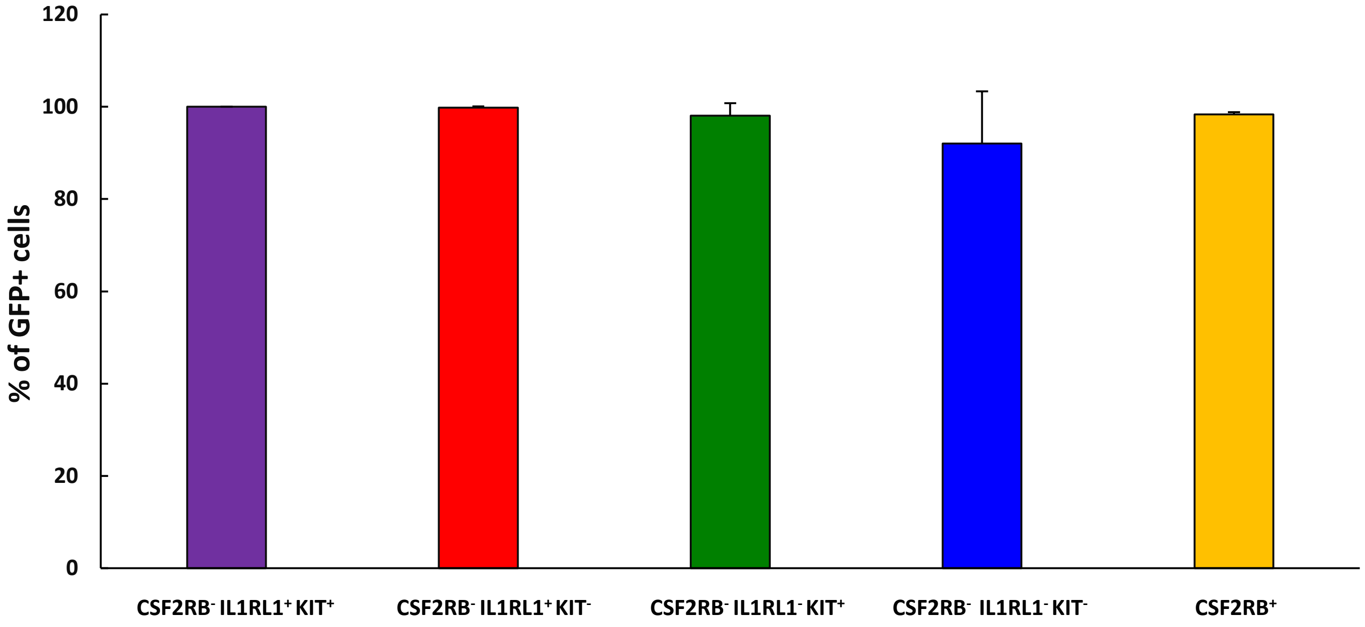

Supplemental Figure S6. GFP expression in each sub-population.

Bar graph showing percentage of GFP^+ cells in each sub-population as indicated. N=2.

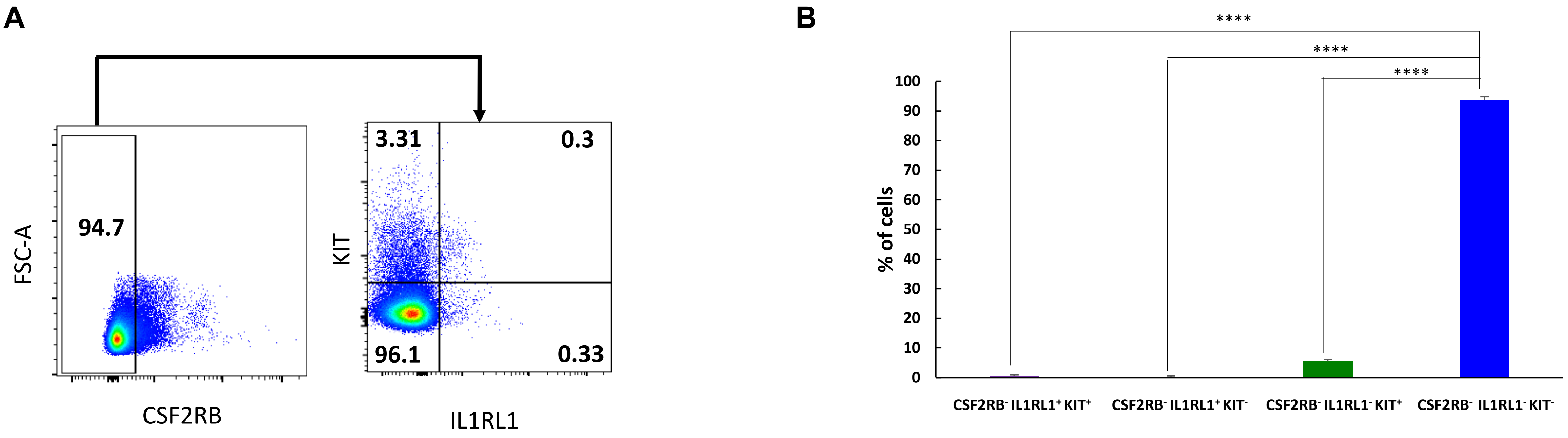

Supplemental Figure S7. Most normal spleen cells are CSF2RB$^-$ IL1RL1$^-$ KIT$^-$.

(**A**) Representative IL1RL1 and KIT staining of CSF2RB$^-$ cells from wild-type mice. (**B**) Bar graph showing IL1RL1 and KIT expression in CSF2RB$^-$ population. N=3; *****P*<.0001.

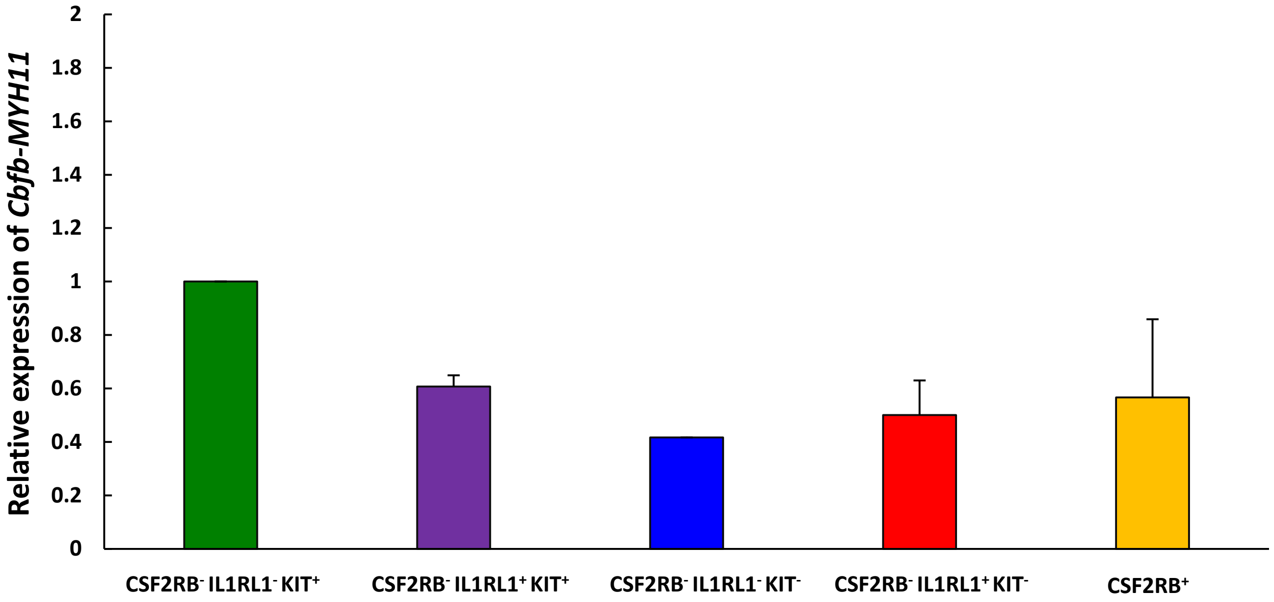

Supplemental Figure S8. Cells from each sub-population do not differ in the expression of *Cbfb-MYH11*.

Bar graph showing the fold changes in mRNA expression of *Cbfb-MYH11* in leukemia cells from *Cbfb-MYH11*$^{+/56M}$, *Mx1-Cre*$^{+}$ mice compared to the relative expression level in CSF2RB^{-} IL1RL1^{-} KIT^{+} population. Relative expression levels were normalized to that of *Actb*. N=2.

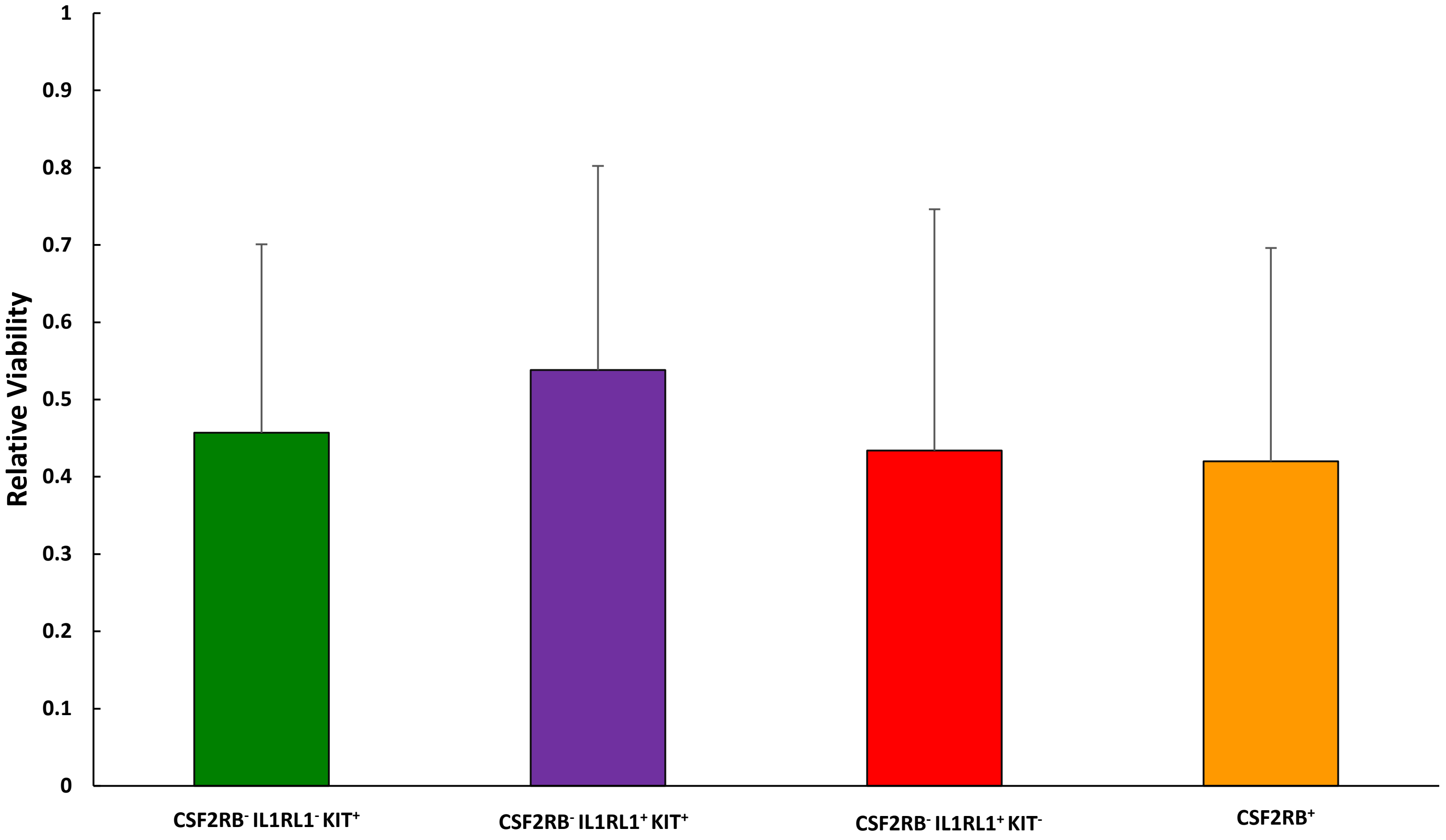

Supplemental Figure S9. Cells from each sub-population have similar viability *in vitro*.

Bar graph showing relative viability of each sorted population after culture on OP-9 cells for 24 hours compared to time zero (0 hour). N=3.

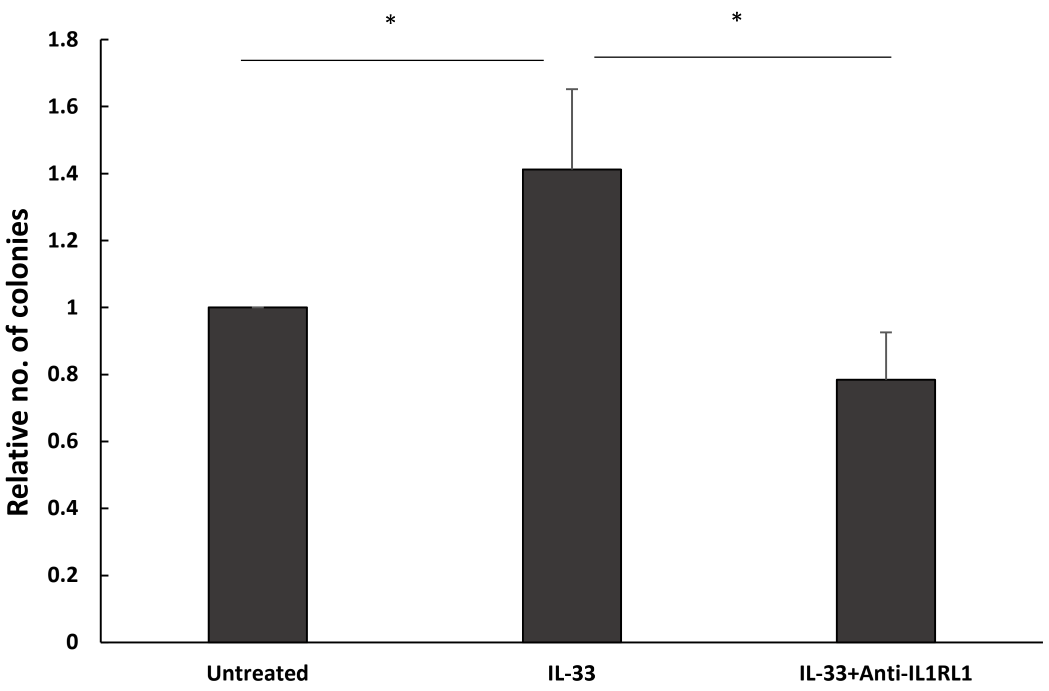

Supplemental Figure S10. IL-33-induced colony-forming ability is dependent on IL1RL1.

Bar graph showing the relative number of colonies observed from equal numbers of *Cbfb-MYH11*$^{+/56M}$, *Mx1-Cre*$^{+}$ leukemia cells cultured in the presence of IL-33 (100 ng/mL) or in combination with an anti-IL1RL1 antibody (1µg/mL) in methylcellulose. Colonies were scored on day 14. N=3. *P<.05

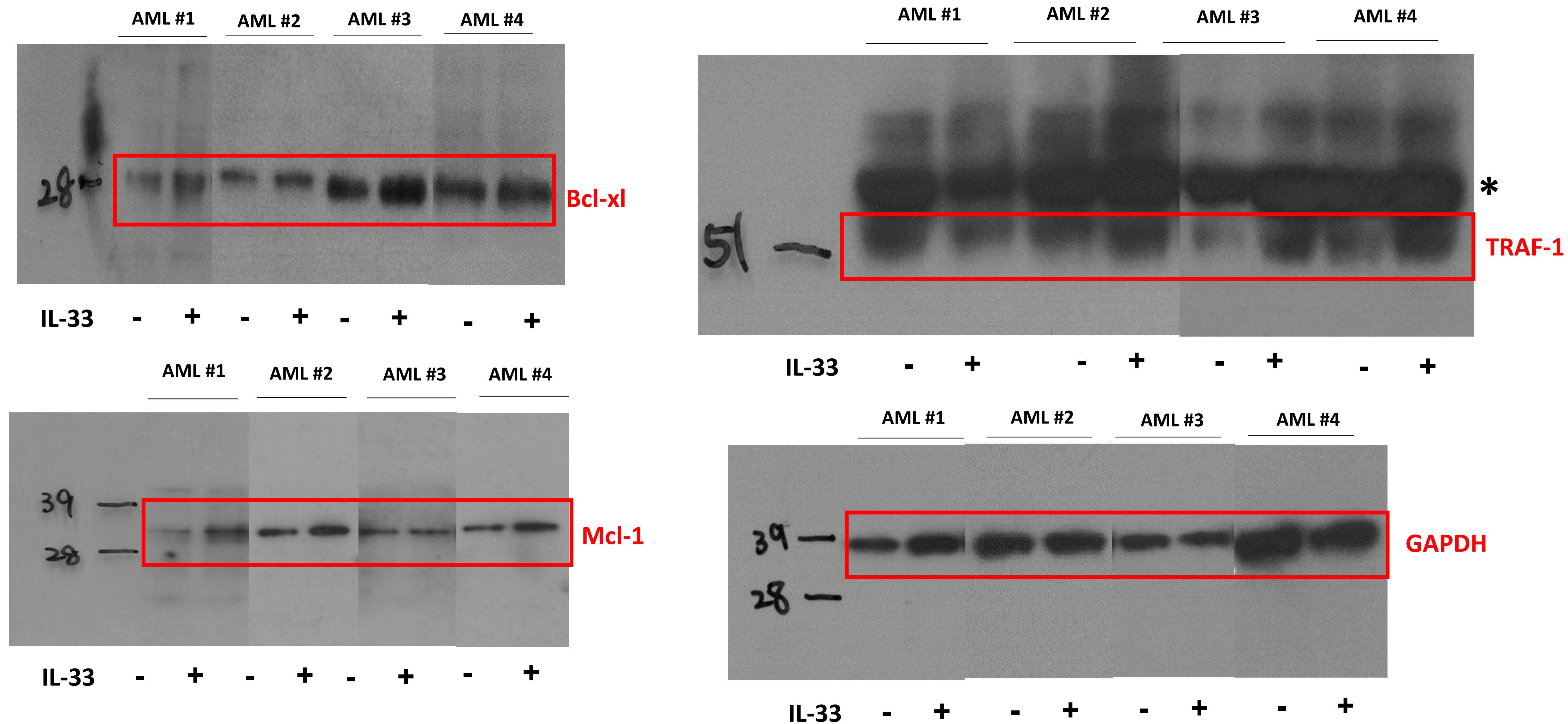

Supplemental Figure S11. Full size images of western blots shown in Fig. 6E.

The cropped area (in red) corresponding to that shown in the main figure. Protein lysates were loaded onto 4-12% Bis-Tris gel and transferred to PVDF membrane. Membranes were cut into 2 fragments and probed using antibodies against Traf-1 (upper right) or Bcl-xl (upper left), stripped and reprobed for Mcl-1 (lower left), and stripped again and reprobed for GAPDH (lower right). * non-specific band at ~64 kDa.

Statistical differences between sub-populations	*P* value (Log-rank test)		
	100 cells	1,000 cells	10,000 cells
CSF2RB$^-$ IL1RL1$^-$ KIT$^+$ **vs.** CSF2RB$^-$ IL1RL1$^+$ KIT$^+$	0.7332	0.0547	**0.0073 ****
CSF2RB$^-$ IL1RL1$^-$ KIT$^+$ **vs.** CSF2RB$^-$ IL1RL1$^-$ KIT$^-$	0.0652	0.0625	0.2336
CSF2RB$^-$ IL1RL1$^-$ KIT$^+$ **vs.** CSF2RB$^-$ IL1RL1$^+$ KIT$^-$	0.4913	0.3272	0.1997
CSF2RB$^-$ IL1RL1$^-$ KIT$^+$ **vs.** CSF2RB$^+$	0.0652	0.6058	0.8843
CSF2RB$^-$ IL1RL1$^+$ KIT$^+$ **vs.** CSF2RB$^-$ IL1RL1$^-$ KIT$^-$	**0.0272***	**0.0008*****	**<0.0001******
CSF2RB$^-$ IL1RL1$^+$ KIT$^+$ **vs.** CSF2RB$^-$ IL1RL1$^+$ KIT$^-$	0.2287	0.3807	0.1262
CSF2RB$^-$ IL1RL1$^+$ KIT$^+$ **vs.** CSF2RB$^+$	**0.0272***	**0.0234***	**0.0075****
CSF2RB$^-$ IL1RL1$^-$ KIT$^-$ **vs.** CSF2RB$^-$ IL1RL1$^+$ KIT$^-$	0.1451	**0.0101***	**0.0257***
CSF2RB$^-$ IL1RL1$^-$ KIT$^-$ **vs.** CSF2RB$^+$	>0.9999	0.1451	0.255
CSF2RB$^-$ IL1RL1$^+$ KIT$^-$ **vs.** CSF2RB$^+$	0.1451	0.1369	0.1737

Supplemental Table S1. Differential survival in mice transplanted with different LSC sub-populations.

Survival differences between groups were compared using the log-rank (Mantle-Cox) test. *P*-values for pairwise comparisons of LSC sub-populations were indicated. N=9; **P* <.05; ***P* <.01; ****P*<.00; *****P*<.0001.

Gene	Primer	Sequence (5'- 3')
Bcl-xl[29]	forward	AAC ATC CCA GCT TCA CAT AAC CCC
	reverse	GCG ACC CCA GTT TAC TCC ATC C
Bcl-2[29]	forward	GTC CCG CCT CTT CAC CTT TCA G
	reverse	GAT TCT GGT GTT TCC CCG TTG G
TRAF-1[29]	forward	GCA GTC ACC CAA GCA CGC CTA C
	reverse	AGC TGG TTC TGT CAG GAG ACA CCC
TRAF-2[29]	forward	CTA CTT GAA TGG CGA CGG CAC TG
	reverse	ACT GCA ACA GAG CAT CAT TGG GG
Mcl-1[41]	forward	GGT GCC TTT GTG GCC AAA CAC TTA
	reverse	ACC CAT CCC AGC CTC TTT GTT TGA
Actb[42]	forward	CCC TAA GGC CAA CCG TGA A
	reverse	CAG CCT GGA TGG CTA CGT ACA
Cbfb-MYH11	forward	GAG AAG GAC ACG CGA ATT TGA AGA TAG
	reverse	CGT GAA GCT GTC TCT GCA GTT G
Runx1[42]	forward	CCA GCA AGC TGA GGA GCG GCG
	reverse	TGA CGG TGA CCA GAG TG

Supplemental Table S2. Mouse Primers for qRT-PCR.
